# The Influence of Medicine upon Character

**Published:** 1892-02

**Authors:** 


### HOMŒOPATHIC PHYSICIAN,

A MONTHLY JOURNAL OF

HOMŒOPATHIC MATERIA MEDICA AND CLINICAL MEDICINE.

"If our school ever gives up the strict inductive method of Hahnemann, we are lost, and deserve only to be mentioned as a caricature in the history of medicine."—CONSTANTINE HERING.

Vol. XII. FEBRUARY, 1892. No. 2.

### EDITORIAL.

THE INFLUENCE OF MEDICINE UPON CHARACTER.—Every profession or occupation in which a man may engage has some effect upon his character; some effect in developing whatever traits of character he may have lying latent.

Of no profession is this more true than medicine. Whatever of cunning or of simplicity; shrewdness or obtuseness; refinement or coarseness; endurance or weakness; honor or dishonor may lie in the man's nature unobserved is sure to be brought to the surface by the trials of medical practice.

The practice of Homœopathy especially taxes the character of its votary to the utmost. Steadfastness of purpose and perseverance in his path of duty notwithstanding the obstacles of prejudice and skepticism; endurance and silence under the lash of persecution, treachery and calumny, are the traits that must form a part of the character of the earnest follower of the Hahnemannian law. These traits *have* been made manifest in the most obvious way in the masters of homœopathic practice who have in the past constituted the "old guard" of our school.

But it is at the bedside of the suffering patient that the qualities which determine the real capacity of the physician for his career become apparent.

To stand at the bedside of a sufferer whose groans and moans bespeak his agony and excite the sympathies of his sorrowing family ; to listen to their entreaties to the doctor to "do something" for the relief of the patient; and, surrounded thus with such an atmosphere of disquieting influences, calmly to watch the development of the case, and make a careful search for the "guiding symptoms" which shall determine the prescription: truly, this is a great test of moral character.

Hav ng selected what is apparently the simillimum of the "sick condition," for the prescriber to stand undaunted in the face of the distressing expressions of suffering on the part of the patient ; to withstand the appeals of the friends, and the cloud of suspicion and distrust that ever hovers over the head of the sincere Hahnemannian, waiting, waiting, waiting for the eagerly desired relief that will surely come if he have given the truly indicated remedy, but which he well knows will be indefinitely postponed if the prescription be *not* the right medicine, what a manifestation of moral courage !

The soldier "seeking the bubble reputation at the cannon's mouth," is not more courageous, not more daring, not more noble and self-sacrificing than is the doctor under these circumstances. The soldier under his trying ordeal becomes a hero and is applauded and loaded with honors and gifts by his admiring countrymen. Is the doctor similarly rewarded? Rarely. *His* exhibition of strong character is too quiet and refined and subtle to receive the notice of even comparatively cultivated people. Too often his work is ignored, his motives impugned, his want of success in achieving a rescue from death denounced, and himself traduced and villified. With the possibility of such a result before his eyes, the danger of the death of his patient ever present, the dark cloud of possible contagion hanging over him, with all these things vividly before his mind, the doctor's conduct when regulated by self-control and self-reliance in such a desperate situation reaches the sublime. Yet it is rarely seen, rarely appreciated.

Not every physician stands the test. Many of the profession fall before it. Losing courage, losing confidence in themselves and their system, they fly to every expedient that despair can suggest, and thus we meet with the wide departures from homœopathic practice which are so injurious to the credit of our school and so demoralizing to the practitioner himself.

The man who is equal to such a test; who never diverges from the path of duty which lies in a straight line before him; who never takes his eyes from the goal he would attain, notwithstanding these powerful influences, becomes stronger and more reliable with every experience. He it is, who, in the long run, takes his place at the head of his profession; becomes a distinguished man, a leader among his brethren, rather than the man who has failed before these tests, resorts to expedients, and attempts to fill the measure of his shortcomings by boasts of his prowess and indulgence in detractions of his neighbor.

Thus the practice of medicine develops character and exposes its deficiencies. It turns the practitioner in upon himself, teaching self-control, self-examination, self-dependence, which with tenacity of purpose make that symmetry of character which is as necessary in a physician as a comprehensive knowledge of materia medica.

